# Performance of the Genotype^® ^MTBDRPlus assay in the diagnosis of tuberculosis and drug resistance in Samara, Russian Federation

**DOI:** 10.1186/1472-6890-9-2

**Published:** 2009-03-10

**Authors:** Vladyslav Nikolayevskyy, Yanina Balabanova, Tatyana Simak, Nadezhda Malomanova, Ivan Fedorin, Francis Drobniewski

**Affiliations:** 1 UK HPA Mycobacterium Reference Unit, Clinical TB and HIV Research Group, Institute of Cell and Molecular Science, Barts and The London School of Medicine, Queen Mary University, 2 Newark Street, London, E1 2AT, UK; 2 Samara Oblast TB Service, 154 Novo-Sadovaya Street, Samara 443068, Russian Federation

## Abstract

**Background:**

Russia is a high tuberculosis (TB) burden country with a high prevalence of multidrug resistant tuberculosis (MDRTB). Molecular assays for detection of MDRTB on clinical specimens are not widely available in Russia.

**Results:**

We performed an evaluation of the GenoType^® ^MTBDRplus assay (HAIN Lifescience GmbH, Germany) on a total of 168 sputum specimens from individual patients at a public health laboratory in Central Russia, as a model of a middle income site in a region with high levels of drug resistance. Phenotypic drug resistance tests (DST) were performed on cultures derived from the same sputum specimens using the BACTEC 960 liquid media system.

Interpretable GenoType^® ^MTBDRplus results were obtained for 154(91.7%) specimens with readability rates significantly higher in sputum specimens graded 2+ and 3+ compared to 1+ (RR = 1.17 95%CI 1.04–1.32). The sensitivity and specificity of the assay for the detection of rifampicin (RIF) and isoniazid (INH) resistance and MDR was 96.2%, 97.4%, 97.1% and 90.7%, 83.3%, 88.9% respectively. Mutations in codon 531 of the *rpoB *gene and codon 315 of the *katG *gene dominated in RIF and INH resistant strains respectively. Disagreements between phenotypical and molecular tests results (12 samples) could be explained by the presence of rare mutations in strains circulating in Russia and simultaneous presence of resistant and sensitive bacilli in sputum specimens (heteroresistance).

**Conclusion:**

High sensitivity, short turnaround times and the potential for screening large numbers of specimens rapidly, make the GenoType^® ^MTBDRplus assay suitable as a first-line screening assay for drug resistant TB.

## Background

Emergence of multidrug resistance tuberculosis (MDRTB, i.e. resistance to at least rifampicin (RIF) and isoniazid (INH) in conjunction with increasing rates of HIV infection worldwide makes the rapid detection of TB drug resistance a key factor in patients' management and care. Rapid (within 1–2 days) diagnosis of MDRTB in clinical specimens allows the commencement of an appropriate TB treatment regimen earlier and helps to prevent transmission of drug resistant TB bacilli.

The WHO estimates current MDRTB rates in new and previously treated cases globally at 2.9% and 15.3% respectively, with 57% of MDRTB cases coming from three high burden countries (China, India, and the Russian Federation) [1]. MDRTB is more expensive to treat and survival rates (especially in HIV-infected persons) are much lower compared to drug-sensitive TB, which poses a particular problem for low- and middle-income countries like Russia where HIV and TB epidemics are converging and access to second- and third line drug therapy is limited [2,3].

More than 90% of *M. tuberculosis *(MTB) strains phenotypically resistant to RIF and INH respectively harbour point mutations in a 81 bp "core" region of *rpoB *gene (RIF), codon 315 of the *katG *gene and/or regulatory region of the *inhA *gene (INH); other molecular mechanisms of INH resistance include mutations in *ahpC-oxyR *intergenic region and other regions of *inhA *and *katG *genes [4-7]. Various molecular techniques, including conventional sequencing, pyrosequencing, real-time PCR and reverse hybridization assays with DNA probes have been proposed recently for detection of mutations associated with drug resistance with the latter methodology successfully realised in a number of in-house and commercial assays [8-11].

Increasing TB and MDRTB rates, particularly in high TB burden countries, require development and implementation of rapid diagnostic systems able to detect MTB and MDRTB in clinical specimens [12,13]. Automated liquid culture systems have significantly shortened turnaround times compared to solid media, but still require isolation of mycobacterial cultures prior to drug resistance testing; implementation of these systems may not be feasible in laboratories in low- to middle income countries with a high TB and drug resistance burden due to infrastructure limitations, lack of resources and appropriately trained personnel [14].

A limited number of commercial assays for testing clinical specimens (sputum) is currently available on the market, including INNO-LiPA Rif.TB (Innogenetics N.V, Ghent, Belgium) and GenoType^® ^MTBDR (HAIN Lifesciences GmbH, Nehren, Germany) [15-17]. The new version of the latter assay (GenoType^® ^MTBDRplus), targeting the *rpoB *gene associated with the resistance to RIF and both genes (*katG *and *inhA*) associated with the resistance to INH has been evaluated mainly on cultures and clinical specimens in various low incidence settings, demonstrating excellent specificity and good concordance with phenotypical DST results [11,18,19]. A recent study demonstrated the feasibility of this assay as a screening tool when applied in a high-volume public health laboratory in a high TB and HIV, but low drug resistance, incidence area [19].

Russia is a high TB burden country with high rates of TB drug resistance and dominance of Beijing strains reported to be associated with MDRTB [1,20,21]. The current official regulations for TB laboratory diagnosis in Russia do not recommend molecular tools for drug susceptibility testing on cultures and clinical specimens. The Samara oblast (Central Russia) is a hot spot of both TB and HIV epidemics with rates of MDR in new TB cases of approximately 20% rising even higher in previously treated cases and the prison sector [20].

We performed an analysis of the performance of the GenoType^® ^MTBDRplus assay (HAIN Lifesciences GmbH, Nehren, Germany) at the Samara Regional TB Reference laboratory, which is a busy public health laboratory serving a population of 3,000,000 people in the Central Russia, as a model of a middle income site in a region with proven high levels of anti-TB drug resistance. Data from this study contributed to the development of WHO policy on the utility of these assays for MDRTB scsreening.

## Methods

### Clinical specimens, microscopy, identification and phenotypic drug susceptibility tests

A total of 168 consecutive patients provided smear-positive sputum samples (79 samples from new cases and 89 from previously treated patients) at two participating sites: Samara Oblast TB Dispensary and Samara Oblast TB Hospital. Baseline demographic data, including date of birth, gender, residence address, and evidence of previous anti-TB treatment was compiled from patients' medical records and entered into an Access database. The study was approved by the Samara Medical University Ethics Committee. The study was conducted in accordance with the STARD principles for diagnostic accuracy studies: all staff were trained in culture and molecular techniques by the authors and product manufacturer, achieved an appropriate level of proficiency and those performing the molecular analyses were blinded to the reference method of culture-based DST using the MGIT 960 and clinical data.

Sputum specimens (1 specimen per patient) were processed using NALC-NaOH decontamination method (NaOH final concentration 1%) as described elsewhere [22]. After centrifugation and supernatant removal, the sediment was resuspended in 1.0 – 1.5 ml of phosphate buffer and used for smear preparation (Ziel-Nielsen staining), culturing and DNA extraction. Smear grading was performed using WHO recommendations [23].

*Mycobacteria *were cultured using BACTEC MGIT960 liquid culture systems (Becton Dickinson, Cockeysville, USA) and conventional Lowenstein-Jensen media and identified by molecular assays (GenoType Mycobacterium CM, HAIN Lifesciences GmbH, Nehren, Germany) according to manufacturer's guidelines. Phenotypic drug susceptibility tests (DST) for resistance to RIF and INH were performed using the BACTEC MGIT960 according to the manufacturer's recommendations and as described elsewhere [24-26]. Where results of *Mycobacteria *culturing and/or DST using the liquid culture system were uninterpretable, isolates (N = 4) were inoculated and subsequently tested for drug resistance using the absolute concentration method on solid (Lowenstein-Jensen) media.

### Detection of mutations associated with drug resistance

Identification of mutations in *rpoB*, *katG*, and *inhA *genes associated with resistance to RIF and INH in all sputum specimens and a proportion (N = 78) of *Mycobacterial *cultures was performed using GenoType^® ^MTBDRplus kits (HAIN Lifesciences GmbH, Nehren, Germany) according to the manufacturer's recommendations. Briefly, crude DNA was extracted from sputum specimens and cultures by heating suspensions in a dry heating block followed by an incubation on ultrasonic bath. PCR (50 μl/tube) was performed using HotStar Taq DNA Polymerase (Qiagen, Crawley, UK). The number of PCR cycles was 30 and 40 for DNA samples extracted from cultures and sputum, respectively. Prior to hybridization, PCR products were analysed in 1.5% agarose gel stained with ethidium bromide.

After hybridization, membrane strips were attached to the evaluation sheet, read and interpreted by an operator (who was blinded to the bacteriological results and vice versa) according to the manufacturer's recommendations.

All laboratory work (microscopy, bacteriological identification, DST and molecular testing) was performed at laboratories of Samara Oblast TB service (Samara, Russian Federation).

## Results

### Smear microscopy, bacteriological identification and phenotypic drug susceptibility tests

All sputum samples were graded depending on AFB count in the specimen according to the WHO recommendations [23] with 64 samples (38.1%) graded 1+; 60 samples (35.7%) 2+; 42 (25.0%) 3+; two samples contained less than 10 AFB in 100 fields (Table 1). Seventy-nine sputum specimens (47.0%) were collected from patients who had not been treated for TB, and remaining samples were from previously treated patients.

**Table 1:** Performance of the GenoType^® ^MTBDRplus assay depending on the concentration of AFB in sputum specimens (N = 168)

**Sputum AFB grading** **OR** **Previous anti-TB treatment**	**Unreadable GenoType^® ^MTBDRplus patterns** **(N = 14)**	**Unusual (double) GenoType^® ^MTBDRplus patterns** **(N = 39)**
1 – 9 AFB/100 fields (n = 2)	1 (50.0%)	0

1+ (n = 64)	11 (17.2%)	21 (32.8%)

2+ (n = 60)	2 (3.3%)	12 (20.0%)

3+ (n = 42)	0	6 (14.3%)

New cases (N = 79)	7 (8.9%)	17 (21.5%)

Previously treated cases (N = 89)	7 (7.9%)	22 (24.7%)

Mycobacterial cultures were isolated from all sputum specimens included in the study. Vast majority of isolates (165, i.e. 98.2%) were identified as *M. tuberculosis *complex using the molecular (GenoType Mycobacterium CM) tests, and three more isolates were correctly identified as *M. kansasii*.

Valid phenotypic DST results were obtained for 161 cultures (157 on liquid media and 4 on solid LJ media; DST tests were not performed on *M. kansasii *isolates). No phenotypic DST results were obtained for four *M. tuberculosis *complex cultures heavily contaminated with fast growing bacterial and/or fungal flora. The number of strains resistant to RIF, INH, and MDR in the test collection was 111, 118, and 110 comprising 69.0%, 73.3%, and 67.7% respectively.

### Detection of mutations associated with RIF and INH resistance using GenoType^® ^MTBDRplus assay on sputum specimens

Readable GenoType^® ^MTBDRplus assay results were obtained for 154 DNA extracts obtained from sputum samples comprising 91.7% of all extracts available for testing. Of these three strips had no TB bands indicating the presence of non-TB *Mycobacteria *(later cultures grown from these specimens were identified as *M. kansasii *using the HAIN CM assay). GenoType^® ^MTBDRplus strips for the remaining 14 samples were unreadable having either no bands at all or very weak/unreadable bands in *rpoB*, *katG *and/or *inhA *sections.

Analysis of test results demonstrated that ability to read and interpret GenoType^® ^MTBDRplus assay results varied depending on the concentration of *Mycobacteria *in sputum samples (Table 1). Generally, better results (96.7%–100.0% readable results) were achieved with sputum samples with higher AFB counts (2+...3+), whilst for samples containing less *Mycobacteria *(1+) the performance of the assay was poorer (50.0% – 82.8%). Molecular assay readability rates were significantly higher in specimens graded 2+ than in specimens graded 1+ (RR = 1.17 95% CI 1.04 – 1.32). There were no differences in sensitivity of the assay between sputum samples collected from new patients and those previously treated for TB.

Mutations conferring resistance to RIF and INH were detected in 107 and 117 DNA samples extracted from sputum, respectively; all DNA samples where RIF resistance was detected also had mutations in *katG *and/or *inhA *genes indicating they were INH resistant, i.e. MDR (Tables 2, 3). In a vast majority of RIF-resistant isolates (N = 101; 94.4%) codon 531 was affected (including five strains where mutations in the codon 531 were combined with mutations in other codons). Mutations in other codons of *rpoB *gene were less common affecting a total of 11 strains (10.3% of all RIF-resistant strains). Mutations associated with INH resistance were more diverse: eighty strains (68.4%) had mutations in *katG *(codon 315) gene only, four strains (3.4%) had mutations in the *inhA *gene only (position (-15) in the *mabA-inhA *promoter), and remaining strains had mutations both in *katG *and *inhA *genes (Table 2). No mutations in the position (-8) of the *mabA-inhA *promoter were identified.

**Table 2:** Variety of mutations associated with RIF and INH resistance (sputum specimens)

**Drug resistance patterns**					**No of strains resistant to relevant drug**	**%**
**RIF resistance pattern (*rpoB *gene)**						
		
*WT probes*		*Mutant probes*			**N = 107**	

Δ8		S531L			93	86.9

Δ8		-			3	2.8

Δ7		H526Y			1	0.9

Δ7		H526D			1	0.9

Δ7,Δ8		-			3	2.8

Δ6		-			2	1.9

Δ3,Δ8		D516V, S531L			1	0.9

Δ3,Δ8		D516V			1	0.9

Δ2,Δ7		-			1	0.9

Δ2,Δ4		-			1	0.9

**INH resistance pattern**						
		
***katG***		***inhA***				
		
*WT probes*	*Mutant probes*	*WT1 probe*	*WT2 probe*	*Mutant probes*	**N = 117**	

Δwt	S315T1	WT	WT	-	68	58.1

Δwt	S315T1	WT	WT	C15T	5	4.3

Δwt	S315T1	WT	Δwt	-	2	1.7

Δwt	S315T1	Δwt	WT	C15T	2	1.7

Δwt	S315T1	Δwt	WT	-	2	1.7

Δwt	S315T1, S315T2	WT	WT	-	1	0.9

WT	-	Δwt	WT	C15T	3	2.6

WT	-	WT	WT	C15T	1	0.9

WT	S315T1	WT	WT	-	11	9.4

WT	S315T1	WT	WT	C15T	16	13.7

WT	S315T1	WT	WT	A16G	1	0.9

WT	S315T1	WT	WT	C15T, A16G	3	2.6

WT	S315T1	WT	Δwt	-	1	0.9

WT	S315T1	Δwt	Δwt	-	1	0.9

Notes: ΔN (N = 2...8) – missing WT probe

WT – all WT probes are present

Δwt – missing WT probe(s)

**Table 3:** Performance of GenoType^® ^MTBDRplus assay in detection of RIF, INH and MDR resistance in sputum specimens (N = 149)

	RIF	INH	MDR	Fully sensitive
Sensitivity (%)	96.2	97.4	97.1	88.2

Specificity (%)	90.7	83.3	88.9	97.4

PPV (%)	96.2	94.8	95.3	90.0

NPV (%)	90.7	90.9	93.0	96.6

All rates were calculated vs phenotypic results (BACTEC MGIT)

NPV – Negative predictive value

PPV – Positive predictive value

Although most specimens produced results with either wild type OR mutant probes being positive on the hybridization strip, in a proportion of specimens (N = 39, 25.3% of all readable GenoType^® ^MTBDRplus results) both mutant and wild types probes were visible ("double patterns"), in *katG *and *inhA *genes only (Tables 1 and 3). According to manufacturer's recommendations these results may be indicative of either the presence of heterogenous strains or mixed populations of *Mycobacteria *in initial sputum specimens and were all interpreted as resistant to the relevant drug.

Double patterns were more common in DNA specimens extracted from sputa with lower concentration of AFB bacilli (Table 1); prevalence of double patterns was significantly higher in specimens graded 1+ compared to specimens graded 2+ and 3+ (18/102 vs 21/64 RR = 1.8 95% CI 1.04 – 3.12).

Comparative analysis performed on 149 pairs of DST results (phenotypical DST failed for two specimens for which molecular data was available) demonstrated good overall agreement between molecular (sputum specimens) and phenotypic DST results with molecular and phenotypic tests being identical (resistant or sensitive) in 141 (94.6%) and 140 (94.0%) specimens for RIF and INH susceptibility respectively (Table 3). Sensitivity and positive predictive values were generally higher (95.3%...97.4%) for detection of resistant strains whereas specificity and negative predictive values were higher (96.6%...97.4%) for detection of sensitive strains indicating that the molecular assay tends to overestimate resistance when applied on direct sputum specimens.

The total number of discrepant results was 17 (8 disagreements for rifampicin resistance and 9 disagreements for isoniazid resistance in a total of 12 strains) (Table 4). In seven cases (comprising respectively 3.8% and 2.7% of all RIF and INH phenotypically resistant isolates) "wild" type hybridization patterns in relevant genes were registered in DNA samples extracted from sputum specimens which then produced resistant *M. tuberculosis *cultures probably indicating the presence of less common mutations not detected by the current version of the MTBDRPlus assay.

**Table 4:** Analysis of disagreements between molecular (sputum specimens and cultures) and phenotypical DST results

	Molecular DST							Phenotypical DST	
			
	Sputum					Culture			
	
ID			GenoType^® ^MTBDRplus pattern						
	RIF	INH	*rpoB*	*katG*	*inhA*	RIF	INH	RIF	INH
007–035	S	S	WT	WT	WT	S	S	R	R
007–060	S	R	WT	Δwt S315T1	WT	S	S	S	S
007–069	R	R	Δ8 S531L	WT	WT, C15T	S	S	R	S
007–086	R	R	Δ7,Δ8	WT S315T1	WT	S	S	S	S
007–088	R	R	Δ8 S531L	Δwt S315T1	WT	S	S	S	R
007–100	S	S	WT	WT	WT	S	S	S	R
007–121	S	R	WT	WT S315T1	WT	R*	R**	R	R
007–127	S	R	WT	WT S315T1	WT	S	S	S	S
007–141	R	R	Δ8 S531L	WT S315T1	Δwt C15T	S	R**	S	S
007–149	S	R	WT	Δwt S315T1	WT	n/a	n/a	R	S
007–153	R	R	Δ8 S531L	Δwt S315T1	WT	S	R***	S	R
007–166	S	S	WT	WT	WT	n/a	n/a	R	R

Notes: ΔN (N = 2...8) – missing WT probe

WT – all WT probes are present

Δwt – missing WT probe(s)

* – GenoType^® ^MTBDRplus pattern Δ8 S531L

** – GenoType^® ^MTBDRplus pattern WT S315T1

*** – GenoType^® ^MTBDRplus pattern Δwt S315T1

"Mutant" *rpoB *gene patterns and "mutant" *katG *and/or *inhA *patterns were registered respectively in four (9.3%) and six (16.7%) DNA samples, from which phenotypically sensitive *M. tuberculosis *strains were then derived. There were no associations between specific types of mutations (or GenoType^® ^MTBDRplus patterns) and disagreements between molecular and phenotypic results (Table 4).

### Detection of mutations associated with drug resistance using GenoType^® ^MTBDRplus assay on culture specimens

To validate results of the GenoType^® ^MTBDRplus assay on sputum samples and address issues related to "double patterns" and discrepancies between molecular (sputum) and phenotypic results, we tested a panel (N = 78) of cultures for RIF and INH resistance using GenoType^® ^MTBDRplus assay (Tables 1 and 4). This panel included 38 of 39 cultures derived from sputum specimens that had produced double patterns and 10 of 12 specimens with disagreements as well as a 30 other randomly selected samples. Readable molecular results were obtained for all specimens.

Comparison of results revealed some disagreements between three sets of data (molecular DST on sputum, molecular DST on culture, phenotypical DST). In a group of isolates phenotypically sensitive to RIF derived from sputum samples identified as resistant using the GenoType^® ^MTBDRplus assay (N = 4), no mutations in *rpoB *gene were found. Similarly, there were no mutations in *katG *and *inhA *genes in four of six phenotypically sensitive to INH isolates derived from "resistant" sputum specimens (one isolate was not tested and one more had mutation in the *katG *gene). The prevalence of "double" hybridization patterns in DNA specimens extracted from cultures was substantially lower compared to those extracted from sputum (25.8% vs 6.4%, RR 4.03 95%CI 1.65 – 9.81). There were no discrepancies in mutations detected in DNA specimens extracted from sputum specimens and cultures derived from these specimens (Table 4).

## Discussion

In the current study we evaluated the performance of the molecular assay (HAIN Lifescience GmbH GenoType^® ^MTBDRplus) for rapid detection of resistance to the most important anti-TB drugs (RIF and INH) on sputum samples in the region with middle TB incidence and high prevalence of MDRTB in Russian Federation (Samara oblast). Until now, within the Russian Federation, there was only limited evidence of applicability of rapid molecular techniques (biochips) for detection of mutations conferring drug resistance directly in clinical specimens [27]; molecular assays for diagnosis of anti-TB drug resistance are not widely available in the Russian Federation, a high TB burden country [1].

Substantial reduction in the time to diagnose drug-resistant TB, the earlier commencement of appropriate therapy and the potential to prevent transmission of drug-resistant strains constitutes the major advantages of these methods. Recent studies demonstrated the feasibility of the MDRTBPlus assay as an effective tool for MDRTB screening in a high TB, and high MDRTB incidence, region and good concordance with phenotypic DST results [11,18,19]. However, rapid DST on clinical samples using molecular tools has (or potentially may have) a number of drawbacks, generally related to the low concentration of bacilli and possible presence of various types of *Mycobacteria *(eg sensitive and resistant ones) in the sputum specimen. The former issue, potentially leading to a problems with an assay sensitivity, has been addressed by incorporation of two stages (nested) PCR or increasing of number of PCR cycles (INNO-LiPA Rif.TB and GenoType^® ^MTBDRplus respectively), but the sensitivity was still low in smear-negative culture-positive samples [11,18,28]. In addition, increase in assay sensitivity achieved by a large number of PCR cycles may lead to increased sensitivity to bacterial and/or amplicon contamination. The sensitivity of the assay may also be affected by a suboptimal selection of DNA probes and targets for the population studies because prevalence of mutations associated with RIF and INH resistance vary in different geographical regions [5,29,30].

In our collection of strains the spectrum of mutations associated with the resistance to RIF and INH (dominance of single mutations in the codon 531 of the *rpoB *gene and codon 315 of the *katG *gene) was similar or close to previously reported on larger populations in Samara and other regions of Russian Federation [20,31-33]. The proportion of drug resistant strains in which no mutations were detected was low (3.8% and 2.7% for RIF and INH resistance) suggesting that the set of the DNA probes used in the GenoType^® ^MTBDRplus assay covers most of the mutations prevailing in the Russian Federation. Previously reported associations between the above mutations and Beijing strains [20,34] suggest that the assay may be potentially useful in other areas outside Russia with a high prevalence of Beijing family isolates (Eastern Europe, China and South-East Asia).

The proportion of interpretable GenoType^® ^MTBDRplus assay results in our study, i.e. sensitivity of the assay for detection of TB bacilli (91.7%) was high but slightly lower compared to that reported previously (96.8% and 98.6% in [19] and [11] respectively), and, contrary to previous publications, lower readability rates were clearly associated with lower AFB grading in smear microscopy results. The performance of the assay on sputum samples with low concentrations of TB bacilli could probably be enhanced through the use of alternative methods of sputum treatment, potentially involving concentration of microorganisms prior to DNA extraction. An increased number of PCR cycles (40 cycles for sputum analysis compared to 30 cycles for cultures) does not resolve this problem completely and increases the sensitivity of the assay to amplicon contamination.

Overall sensitivity of the GenoType^® ^MTBDRplus assay for detection of RIF, INH and multidrug resistance was high at 96.2%, 97.4%, and 97.1% respectively, which is similar to previously reported results in studies from South Africa, Germany, and Italy [11,18,19] supporting the use of this assay for MDRTB screening. Specificity and negative predictive values were 90.7%, 83.3%, and 88.9% for rifampicin, isolniazid, and MDR respectively suggesting that the molecular assay slightly overestimates drug resistance as defined by phenotypic DST on cultures derived from relevant sputum specimens. We believe that these discrepant results (10 disagreements in total), as well as "double patterns" in a proportion of strips (25.8%) could be explained by a "heteroresistance", i.e simultaneous presence of both drug resistant and sensitive TB bacilli in clinical samples.

We hypothesized that in these cases initial sputum samples contained mixtures of resistant and sensitive bacilli and, whilst mutant genotypes were recognized by the molecular assay (therefore masking sensitive genotypes) the sensitive bacilli might have grown preferentially on liquid media giving "sensitive" phenotypic DST results.

Heteroresistance, initially reported by Rinder et al. [35], is an important factor which can affect the accuracy and reliability of drug susceptibility testing using clinical specimens, because phenotypic results after isolation of pure cultures may not be representative of the initial mixture of *Mycobacteria *in the sputum. This usually underestimated phenomenon complicates interpretation of diagnostic assay results and may have been a reason for discordant results and "double patterns" (positive hybridization with mutant and wild type probes) on GenoType^® ^MTBDR and GenoType^® ^MTBDRplus membranes in our and recent studies [15,18]. We assume that heteroresistance is more likely to occur in high TB incidence areas and in cultures isolated from chronic patients as they have more opportunity to become infected with various populations of *Mycobacteria *[36]; therefore "double patterns" were more common in our collection of samples obtained in high TB drug resistance region. Heteroresistance should be further investigated by molecular fingerprinting of clinical specimens (eg PCR-based VNTR typing) and detection of mutations in artificial "spiked" sputum specimens to establish mechanisms of preferential recognition of certain genotypes present in the specimen.

## Conclusion

The GenoType^® ^MTBDRplus assay is a sensitive and specific tool for diagnosis of RIF, INH resistance and MDR in sputum specimens. The short turnaround times and the potential for rapid screening of large numbers of specimens make it suitable as a first-line screening assay for TB drug resistance

## Authors' contributions

VN participated in the study design, carried out molecular tests, participated in data analysis and drafted the manuscript; YB participated in the study design, data analysis and helped to draft manuscript; TS carried out molecular and bacteriological tests; NM carried out bacteriological tests; IF participated in the study design and co-ordination and helped to draft manuscript; FD co-ordinated the study, participated in data analysis and drafting the manuscript. All authors read and approved the final version of the manuscript.

## Pre-publication history

The pre-publication history for this paper can be accessed here:


